# Supplementary material for: MEN1 deficiency leads to neuroendocrine differentiation of lung cancer and disrupts the DNA damage response
Source: Nat Commun. 2020 Feb 21;11:1009. doi: 10.1038/s41467-020-14614-4 (PMC7035285; doi:10.1038/s41467-020-14614-4)
Supplement: Supplementary file 1 — Supplementary Information [file 41467_2020_14614_MOESM1_ESM.pdf]

Supplemental information

MEN1 deficiency leads to neuroendocrine differentiation of lung cancer and disrupts the DNA damage response

Huan Qiu, Bang-Ming Jin, Zhan-Feng Wang, Bin Xu, Qi-Fan Zheng, Li Zhang, Ling-Yu Zhu, Shuang Shi, Jun-Bo Yuan, Xiao Lin, Shu-Bin Gao, Guang-Hui Jin

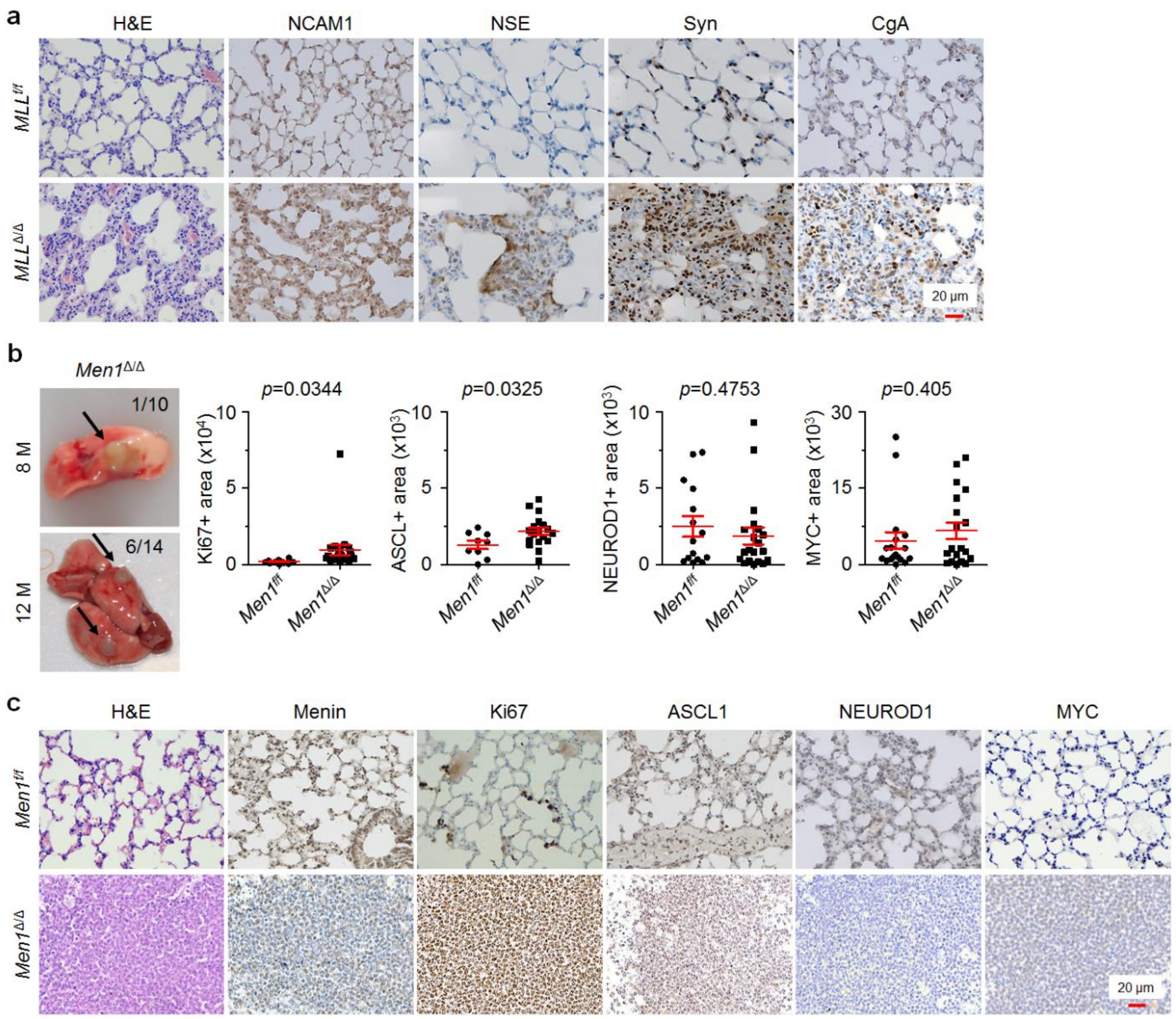

Supplementary Figure 1 (Related to Figure 1): Inactivation of menin/MLL results in NE differentiation of lung cancer. a Representative H&E and IHC staining in the lung tissues of *MLL*$^{f/f}$ mice (*n*=21) and *MLL*$^{Δ/Δ}$ mice (*n*=16) at 4 months post TAM treatment. Scale bars, 20 μm. **b** Brightfield images of lung tissues dissected from *Men1*$^{Δ/Δ}$ mice after TAM treatment for 8 (*n*=20) or 12 (*n*=14) months (left). Tumors on the lung surface are indicated with black arrows; Automatic quantification of Ki67, ASCL1, NEUROD1 and MYC IHC staining in *Men1*$^{f/f}$ (*n*=18) and *Men1*$^{Δ/Δ}$ (*n*=20) 8 months mice lung tissues. **c** Representative H&E and IHC staining for the indicated antibodies in lung tissues from *Men1*$^{f/f}$ and *Men1*$^{Δ/Δ}$ mice at 8 months post TAM treatment. Scale bars, 20 μm. Data are represented as mean ± SEM in **b**. Dots in **b** depict individual samples. Significance determined by Two-tailed unpaired t tests in **b** are indicated. Source Data are provided as a Source Data file.

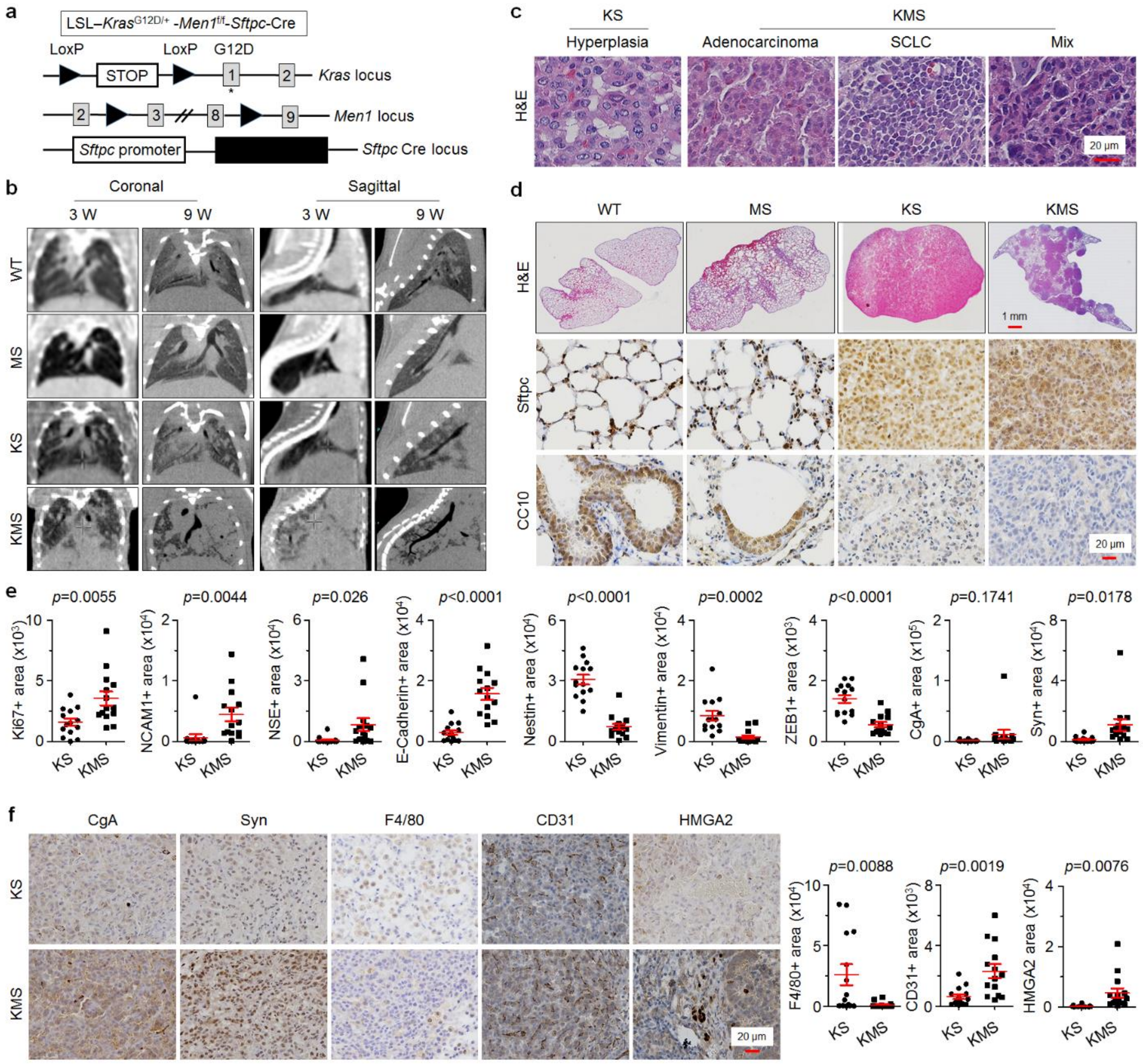

Supplementary Figure 2 (Related to Figure 2): Loss of *Men1* dramatically accelerates *Kras* mutation-induced lung carcinogenesis. a Schematic representation of the modified alleles at the *Kras*^{G12D}, *Men1*, and *Sftpc* loci. The three mouse genotypes were crossed to generate *LSL-Kras*$^{G12D/+}$; *Men1*$^{f/f}$; *Sftpc*-Cre (KMS), *LSL-Kras*$^{G12D/+}$; *Sftpc*-Cre (KS), and *Men1*$^{f/f}$; *Sftpc*-Cre (MS) models, with WT mice as controls. Combined modification of *Kras*^{G12D} and *Men1* conditional alleles after Cre recombinase expression was controlled by TAM treatment. The triangles represent flox sites. The rectangles indicate exons. **b** Coronal and sagittal thoracic microCT images of mice of the indicated genotypes at 3 and 9 weeks after TAM injection. **c** Representative H&E staining showing the different pathological types of lung tumors derived from KS and KMS mice at 9 weeks. Scale bars, 20 µm. **d** Representative H&E and IHC staining for Sftpc and CC10 in lung sections derived from the indicated mice at 11 weeks. Scale bars, 1 mm (H&E) and 20 µm (IHC). **e** Automatic quantification of

Ki67, NCAM1, NSE, E-cadherin, ZEB1, Nestin, Vimentin (related to Fig. 2e) CgA, Syn (related to Supplementary Fig. 2f) IHC staining in KS (*n*=14) and KMS (*n*=14) mice lung tissues. **f** Representative IHC staining for CgA, Syn, F4/80, CD31, and HMGA2 in lung tumors from KS and KMS mice. Scale bars, 20 μm (IHC). Automatic quantification of F4/80, CD31, HMGA2 IHC staining in KS (*n*=14) and KMS (*n*=14) mice lung tissues. Data are represented as mean ± SEM in **e** and **f**. Dots in **e** and **f** depict individual samples. Significance determined Two-tailed unpaired t tests in **e** and **f** are indicated. Source Data are provided as a Source Data file.

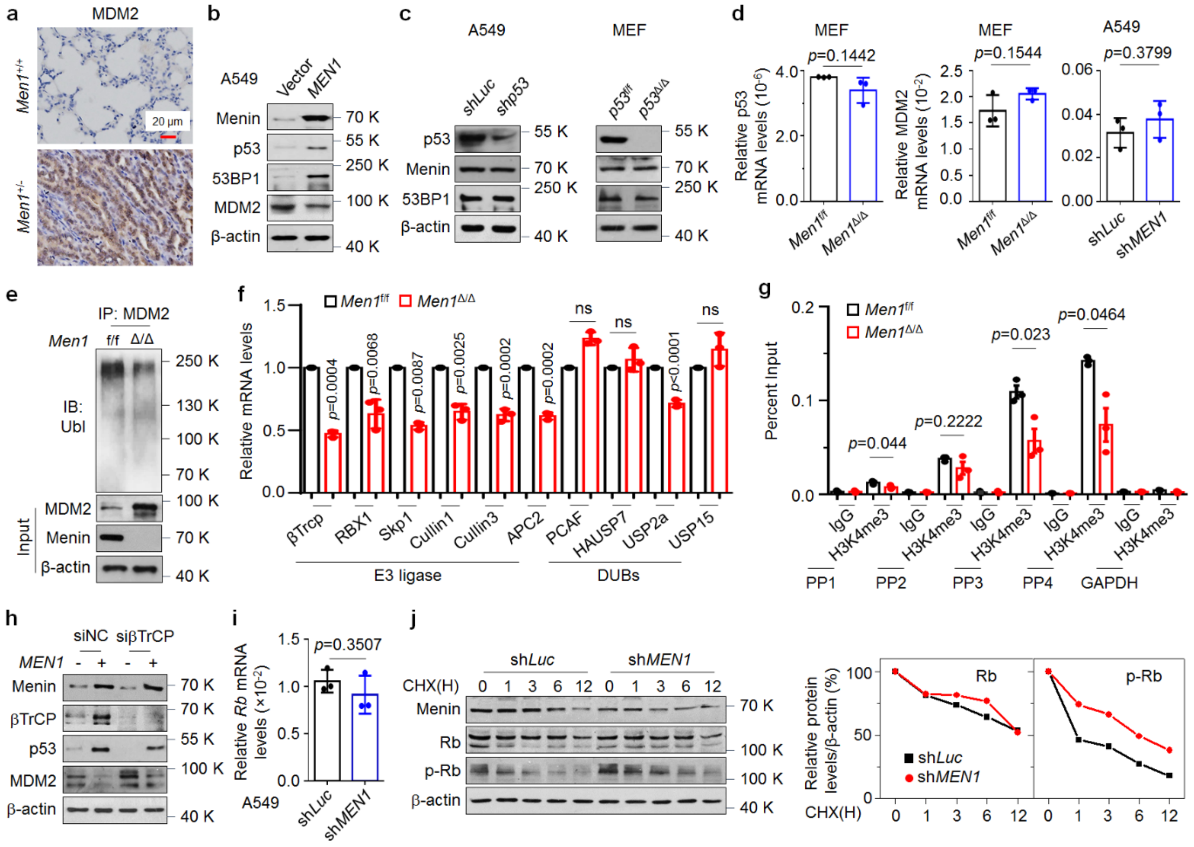

Supplementary Figure 3 (Related to Figure 3): Loss of *Men1* leads to inactivation of p53 and the Rb pathway. a IHC staining was performed with specific MDM2 antibody in lung sections from the *Men1*$^{+/+}$ and *Men1*$^{+/-}$ mice at 18 months (from Figure 1). Scale bars, 20 μm. **b-c** Western blotting was used to detect the expression of the indicated proteins in A549-vector and A549-*MEN1* cells (**b**), A549-sh*Luc* and A549-sh*MEN1* cells, and *p53*$^{f/f}$ and *p53*$^{\Delta/\Delta}$ MEF cells (**c**). **d** RT-qPCR was used to detect the mRNA expression of *p53* and *MDM2* in *Men1*$^{f/f}$ and *Men1*$^{\Delta/\Delta}$ MEF cells and in A549-sh*Luc* and A549-sh*MEN1* cells. **e** Co-IP with an anti-MDM2 antibody and detection of ubi-MDM2 with an anti-ubiquitin antibody were performed via Western blotting of samples from *Men1*$^{f/f}$ and *Men1*$^{\Delta/\Delta}$ MEF cells treated with 25 μg/ml MG132 for 6 hours. **f** RT-qPCR was used to detect the mRNA expression of E3 ligase and DUB genes in *Men1*$^{f/f}$ and *Men1*$^{\Delta/\Delta}$ MEF cells. **g** ChIP-PCR with an anti-H3K4me3 antibody was used to detect the enrichment of H3K4me3 at the indicated promoter regions in *Men1*$^{f/f}$ and *Men1*$^{\Delta/\Delta}$ MEF cells. IgG served as the negative control. **h** Western blotting was used to detect the expression of the indicated proteins in A549-Vector and A549-*MEN1* cells transfected with siNC or siβTrCP for 48 hours. **i** RT-qPCR was used to detect the mRNA expression of *Rb* in A549-sh*Luc* and A549-sh*MEN1* cells. **j** A549-sh*Luc* and A549-sh*MEN1* cells were treated with 50 μg/ml

cyclohexamide (CHX), and the expression of menin, Rb, and p-Rb was determined by Western blotting (left). The relative expression of Rb or p-Rb was quantified by gray scanning, and the protein levels at the indicated time points are presented relative to those at 0 min (right). Data are represented as mean ± SD in **d**, **f**, and **i** and as mean ± SEM in **g**. Dots in **d**, **f**, **g** and **i** depict one repeat. Significance determined by Two-tailed unpaired t tests in **d**, **f**, **g** and **i** are indicated. Results in **b**, **c**, **d**, **e**, **f**, **g**, **h**, **i**, and **j** are representative of three independent experiments. Source Data are provided as a Source Data file.

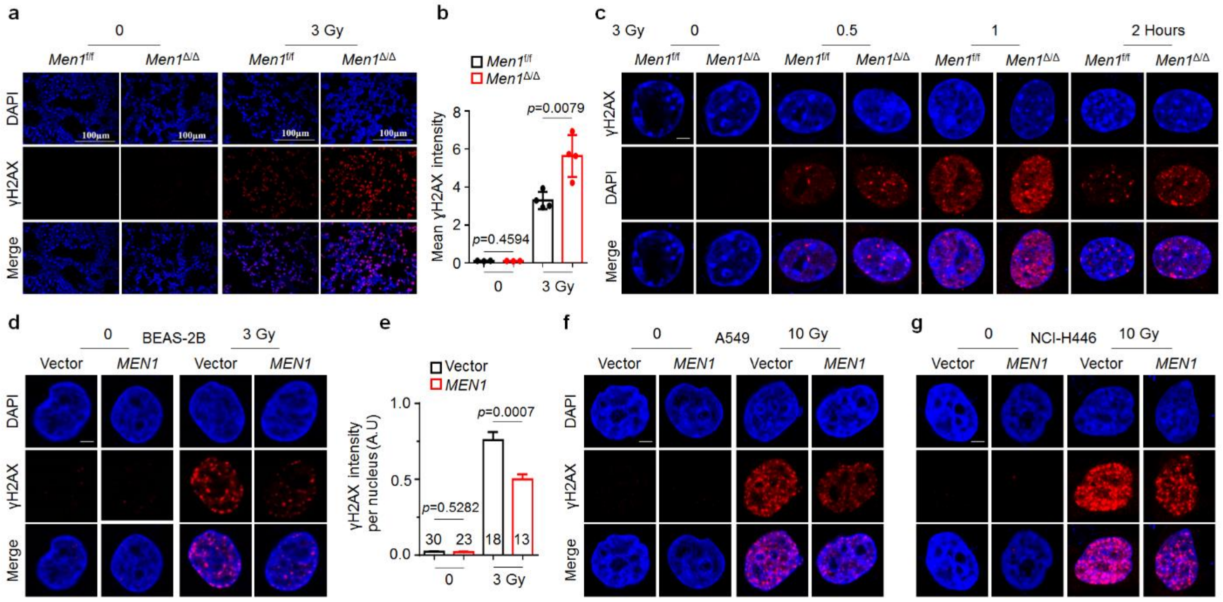

Supplementary Figure 4 (Related to Figure 4): Loss of *Men1* triggers aberrant DNA damage response. a Representative IF staining of γH2AX (red) and DAPI (blue) in lung tissues from *Men1*$^{f/f}$ and *Men1*$^{\Delta/\Delta}$ mice 1 hour after whole-body exposure to 3 Gy of IR. Scale bars, 100 μm. **b** Quantification of γH2AX intensity shown in (**a**). *Men1*$^{f/f}$ mice (*n*=3), *Men1*$^{\Delta/\Delta}$ mice (*n*=4). **c** IF staining for γH2AX (red) and DAPI (blue) in *Men1*$^{f/f}$ and *Men1*$^{\Delta/\Delta}$ MEF cells at the indicated time points after exposure to 3 Gy of IR, Scale bars, 5 μm. **d** IF staining for γH2AX (red) and DAPI (blue) in BEAS-2B-vector and BEAS-2B-*MEN1* cells 1 hour after exposure to 3 Gy of IR. Scale bars, 5 μm. **e** Quantification of γH2AX intensity shown in (**d**). **f-g** IF staining for γH2AX (red) and DAPI (blue) in A549-vector and A549-*MEN1* cells, NCI-H446-vector and NCI-H446-*MEN1* cells 1 hour after exposure to 10 Gy of IR. Scale bars, 5 μm. Data are represented as mean ± SD in **b** and as mean ± SEM in **e**. Dots in **b** depict individual samples. Significance determined by Two-tailed unpaired t tests in **b** and Mann-Whitney U tests in **e** are indicated. Results in **a**, **c**, **d**, **f**, and **g** are representative of three independent experiments. Source Data are provided as a Source Data file.

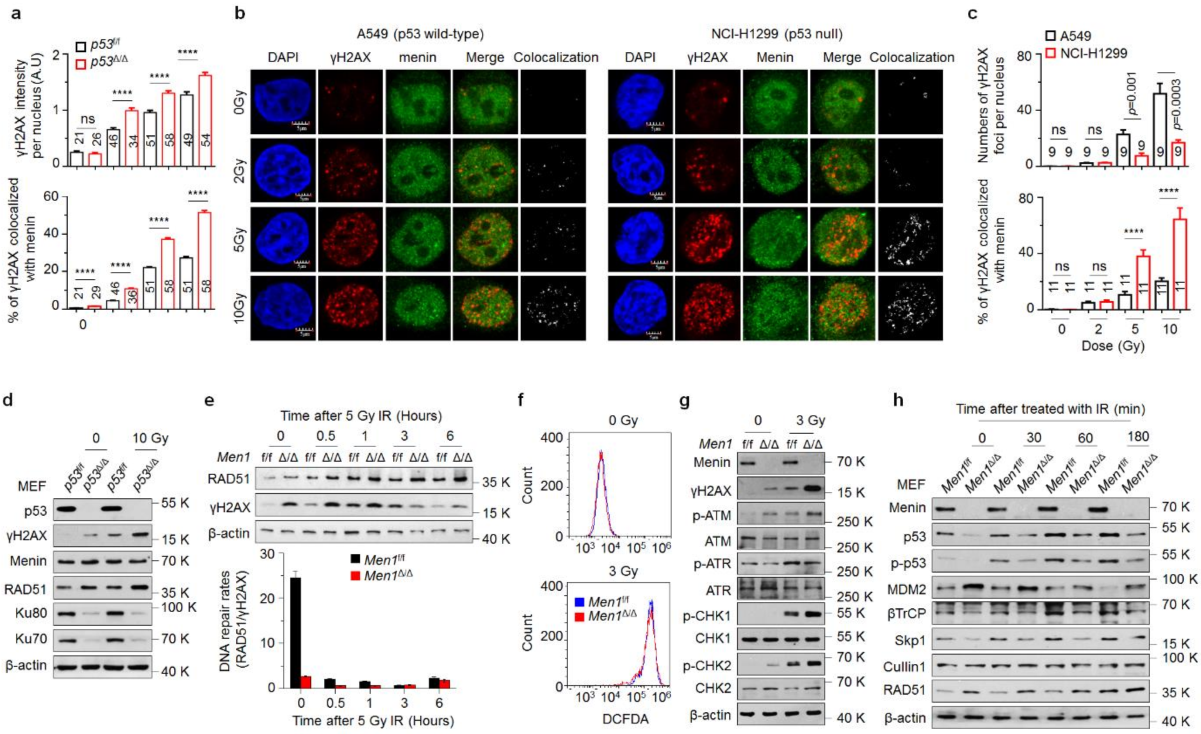

Supplementary Figure 5 (Related to Figure 4): Loss of *Men1* triggers aberrant DNA damage response. a Quantification of the γH2AX foci per nucleus and the percentage of menin colocalized with γH2AX per nucleus shown in Fig. 4d. **b** IF staining for γH2AX (red), menin (green) and DAPI (blue) in A549 and NCI-H1299 cells 1 hour after exposure to the indicated IR doses. Scale bars, 5 µm. **c** Quantification of the γH2AX foci per nucleus and the percentage of menin colocalized with γH2AX per nucleus shown in (**b**). **d** Western blotting was used to detect the expression of the indicated proteins in *p53*$^{f/f}$ and *p53*$^{\Delta/\Delta}$ MEF cells 1 hour after exposure to 10 Gy of IR. **e** Western blotting was used to detect the expression of the indicated proteins in *Men1*$^{f/f}$ and *Men1*$^{\Delta/\Delta}$ MEF cells at the indicated time points after exposure to 5 Gy of IR (top). The relative expression of RAD51 or γH2AX was quantified by gray scanning, and DNA repair rates are calculated by RAD51/γH2AX (bottom). **f** Flow cytometry was used to detect the production of ROS in the *Men1*$^{f/f}$ and *Men1*$^{\Delta/\Delta}$ MEF cells stained with 20 µM DCFDA, 6 hours after exposure to 3 Gy of IR. **g** Western blotting was used to detect the expression of the indicated proteins in *Men1*$^{f/f}$ and *Men1*$^{\Delta/\Delta}$ MEF cells 1 hour after exposure to 3 Gy of IR. **h** Western blotting was used to detect the expression of the indicated proteins in *Men1*$^{f/f}$ and *Men1*$^{\Delta/\Delta}$ MEF cells at the indicated time points after exposure to 10 Gy of IR. Data are represented as mean ± SEM in **a**, **c** and as mean ± SD in **e**. Significance determined by Mann-Whitney U tests in **a** and **c** are

indicated. Results in **b**, **d**, **e**, **f**, **g**, and **h** are representative of three independent experiments. Source Data are provided as a Source Data file.

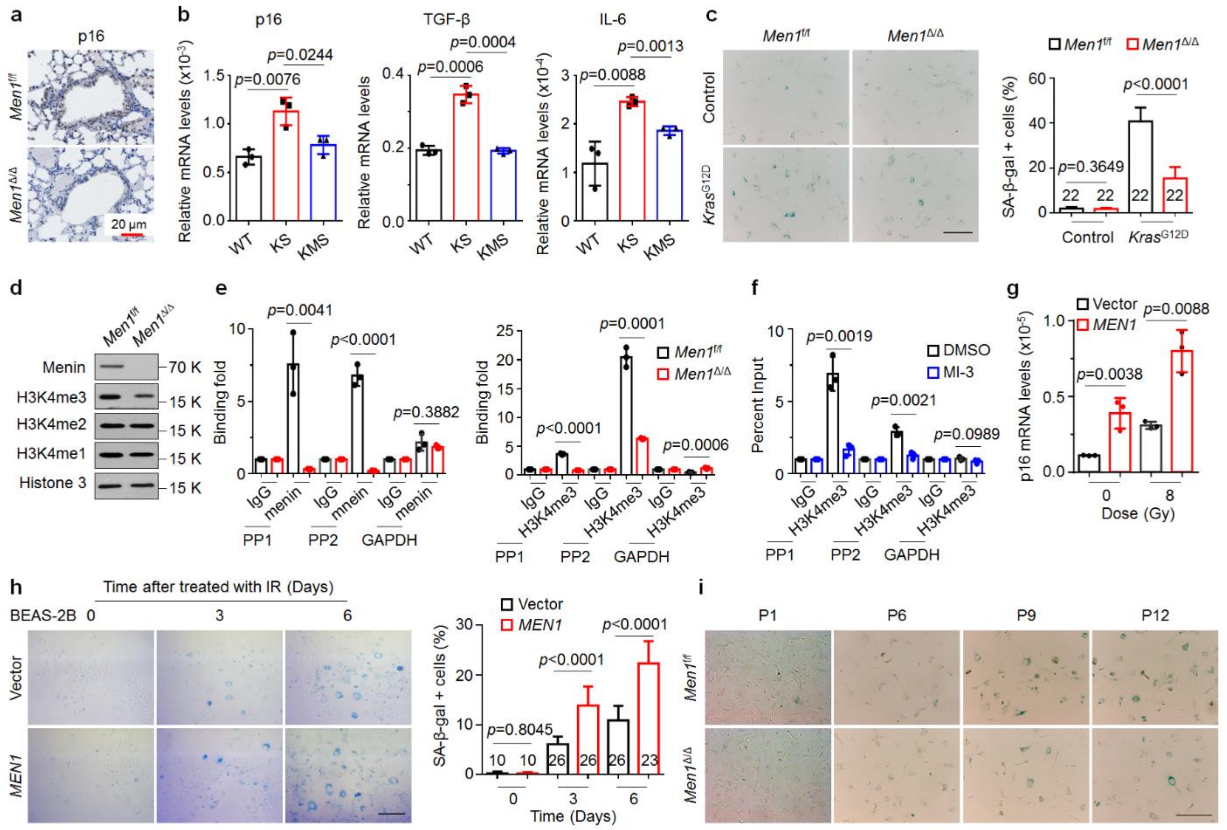

Supplementary Figure 6 (Related to Figure 5): Menin is required for stress-induced senescence programs. a IHC staining of p16 in lung sections from *Men1*$^{f/f}$ and *Men1*$^{\Delta/\Delta}$ mice (8 months). Scale bars, 20 μm. **b** RT-qPCR was used to detect the mRNA expression of *p16*, *TGF-β* and *IL-6* in lung tissues from WT, KS and KMS mice (11 weeks). **c** SA-β-gal staining of primary *Men1*$^{f/f}$ and *Men1*$^{\Delta/\Delta}$ MEF cells (left) and quantification of SA-β-gal-positive cells (right). Primary MEF cells (passage 3) were induced with 1 μM TAM and subsequently infected with retrovirus particles expressing *Kras*^{G12D} for 2 days. Cells infected with pLNCX2 plasmid served as controls. Scale bars, 100 μm. **d** Histone extracts were subjected to western blotting to determine H3K4me1, H3K4me2, and H3K4me3 levels in primary *Men1*$^{f/f}$ and *Men1*$^{\Delta/\Delta}$ MEF cells at passage 3 after induction with 1 μM TAM. **e** ChIP-qPCR was performed with anti-menin and anti-H3K4me3 antibodies to assess the enrichment of menin and H3K4me3 at the *p16* promoter regions in *Men1*$^{f/f}$ and *Men1*$^{\Delta/\Delta}$ MEF cells. IgG served as the negative control. **f** ChIP-qPCR was performed with an anti-H3K4me3 antibody to assess the enrichment of H3K4me3 at the *p16* promoter regions in IMR-90 cells treated with or without MI-3 (10 μM) for 2 days. IgG served as the negative control. **g** RT-qPCR was used to detect the mRNA expression of *p16* in BEAS-2B-vector and BEAS-2B-*MEN1* cells 3 days after exposure to 8 Gy of IR. **h** SA-β-gal staining of

BEAS-2B-vector and BEAS-2B-*MEN1* cells at the indicated time points after exposure to 8 Gy of IR (left) and quantification of SA-β-gal-positive cells (right). Scale bars, 100 μm. **i** SA-β-gal staining of primary *Men1*$^{f/f}$ and *Men1*$^{\Delta/\Delta}$ MEF cells. After induction with 1 μM TAM, primary MEF cells were serially passaged, and SA-β-gal staining was performed at passages 1, 6, 9, and 12. Scale bars, 100 μm. Data are represented as mean ± SD in **b**, **c**, **g**, **h,** and as mean ± SEM in **e** and **f**. Dots in **b**, **e**, **f** and **g** depict one repeat. Significance determined by Two-tailed unpaired t tests in **b**, **c**, **e**, **f, g** and **h** are indicated. Results in **b**, **d**, **e**, **f**, and **i** are representative of three independent experiments. Source Data are provided as a Source Data file.

Supplementary Table 1 (Related to Figure 6): Correlation analysis of menin expression and clinicopathological features in NSCLC.

group	menin expression			
	**All cases**	**high**	**low**	***p* value**
**Age（years）**				p=0.038[a]
≤58.9	72	47(65.3%)	25 (34.7%)	
>58.9	85	68 (80%)	17 (20%)	
**Sex**				p=0.792[a]
Male	87	63 (72.4%)	24 (27.6%)	
Female	70	52 (74.2%)	18 (25.8%)	
**Tumor types**				p=0.440[a]
adenocarcinoma	119	89 (74.8 %)	30 (25.2%)	
others	38	26 (68.4%)	12 (31.6%)	
**survival** (median)		58.5 mo	29 mo	
**survival** (average)		41.3 mo	22.1 mo	

a: Chi-square test, two sided.

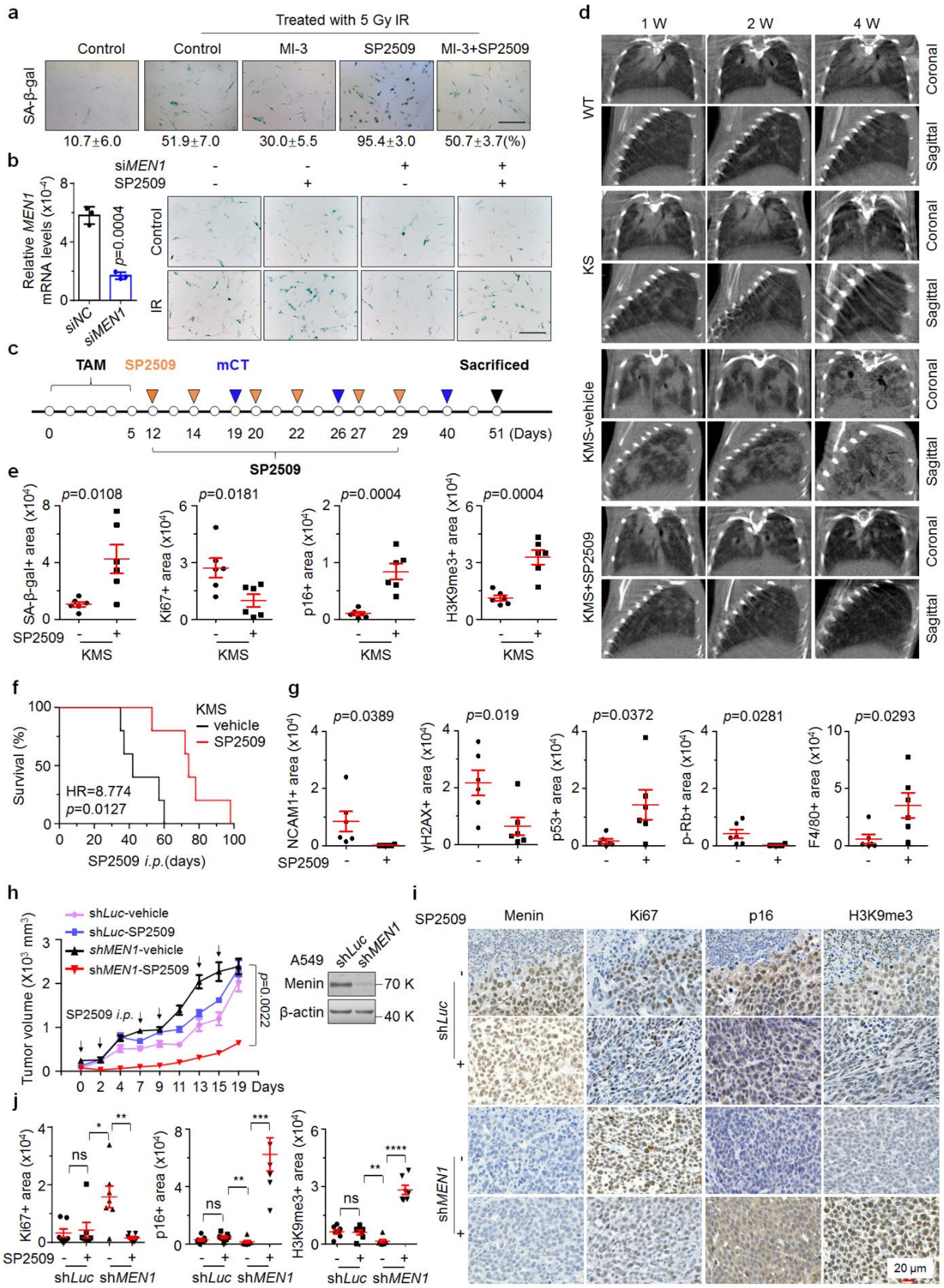

Supplementary Figure 7 (Related to Figure 7): An LSD1 inhibitor reverses NE differentiation induced by loss of menin. a IMR-90 cells were not treated or were treated with MI-3 (10 μM) for 48 hours. Beginning at 44 hours, one group of cells was

also treated with SP2509 (1 μM) for 4 hours before being exposed to 5 Gy of IR and collected after 48 hours. Scale bars, 100 μm. **b** RT-qPCR was used to detect the mRNA levels of *MEN1* in IMR-90 cells 48 hours after si*MEN1* transfection (left). Representative images of SA-β-gal staining in IMR-90 si*MEN1* cells treated with SP2509 followed by 5 Gy of IR (right). Scale bars, 100 μm. **c** Schematic representation of the drug studies performed *in vivo*. One week after induction with 100 mg/kg TAM, WT, KS, and KMS mice were randomly assigned to receive 25 mg/kg SP2509 twice a week for 3 weeks and were imaged by microCT at 1, 2 and 4 weeks after treatment. **d** Coronal and sagittal microCT images in the indicated planes from WT, KS, KMS-vehicle, and SP2509-treated KMS mice at 1, 2 and 4 weeks after treatment. Vehicle, corn oil containing 10% DMSO. **e** Automatic quantification of SA-β-gal and IHC staining in KMS-vehicle (*n*=6) and KMS-SP2509 (*n*=6) mice consecutive lung tissues sections (related to Fig. 7g). **f** Kaplan-Meier survival analysis for vehicle and 25 mg/kg SP2509 *i.p* treated KMS mice. **g** Automatic quantification of NCAM1, p53, p-Rb, γH2AX, and F4/80 IHC staining in KMS-vehicle (*n*=6) and KMS-SP2509 (*n*=6) mice lung tissues. **h** The A549 cells were stably transfected with either sh*Luc* or sh*MEN1* retrovirus, and Western blotting was used to detect the expression of menin; Xenograft tumor growth curve (*n*=7 for per group). The arrows indicate the time points at which SP2509 treatment. **i** IHC staining for menin, Ki67, p16, H3K9me3 in the indicated groups, Scale bars, 20 μm. **j** Automatic quantification of IHC staining in indicated xenograft tumor sections (related to supplementary Fig. 7i). *n*=7 per group. Scale bars, 20 μm. Data are represented as mean ± SD in **a**, **b**, **e**, **g**, and **j**. Dots in **b** depict one repeat and in **e**, **g** and **j** depict individual samples. Significance determined by Two-tailed unpaired t tests in **b**, **e**, and **j** are indicated. The Hazard Ratios (HR) and *p*-values by log-rank (Mantel-Cox) test are indicated in **f**. *$p<0.05$, **$p<0.005$, ****$p<0.0001$. Results in **a**, **b**, and **h** are representative of three independent experiments. Source Data are provided as a Source Data file.

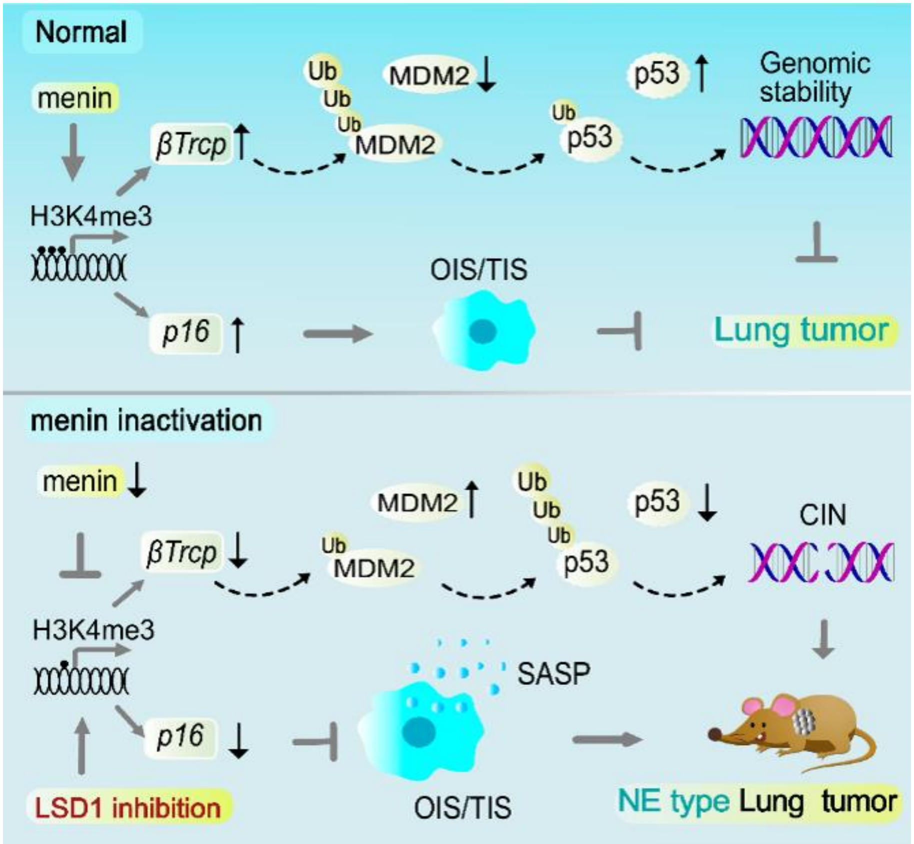

Supplementary Figure 8: *Men1* deletion triggers genomic instability and disordered senescence programs to induce NE differentiation in lung cancer. Under physiological conditions, menin-mediated H3K4me3 modification regulates βTrCP and maintains stability of MDM2/p53 protein, which in turn maintaining genomic stability and preventing lung cancer. menin-βTrCP-MDM2/p53 signaling pathway axis was disrupted by *Men1* deletion, which degraded p53 protein through reducing the ubiquitination of MDM2, which leads to gene mutation and DNA damage accumulation. Meanwhile, loss of *Men1* disrupted senescence programs by downregulating p16 or SASP pathway. Cells with DNA damage bypass the onsets of senescence to induce NE differentiation of lung cancer, which was reversed by LSD1 inhibitors.

Supplemental Table 2. Library of epigenetics small molecular compounds

Catalog Number	Product Name	Target
S1030	Panobinostat (LBH589)	HDAC
S1045	Trichostatin A (TSA)	HDAC
S1047	Vorinostat (SAHA, MK0683)	HDAC
S1053	Entinostat (MS-275)	HDAC
S1085	Belinostat (PXD101)	HDAC
S1090	PCI-24781 (Abexinostat)	HDAC
S1095	LAQ824 (Dacinostat)	HDAC
S1096	Quisinostat (JNJ-26481585)	HDAC
S1122	Mocetinostat (MGCD0103)	HDAC
S1194	CUDC-101	HDAC, EGFR, HER2
S1200	Decitabine	DNA/RNA Synthesis
S1216	PFI-1 (PF-6405761)	Others
S1396	Resveratrol	Sirtuin
S1422	Droxinostat	HDAC
S1484	MC1568	HDAC
S1515	Pracinostat (SB939)	HDAC
S1541	EX527 (Selisistat)	Sirtuin
S2012	PCI-34051	HDAC
S2170	Givinostat (ITF2357)	HDAC
S2244	AR-42	HDAC
S2391	Quercetin	PI3K, PKC, Src, Sirtuin
S2627	Tubastatin A HCl	HDAC
S2693	Resminostat	HDAC
S2759	CUDC-907	HDAC, PI3K
S2779	M344	HDAC
S2804	Sirtinol	Sirtuin
S2818	CI994 (Tacedinaline)	HDAC
S2821	RG108	Transferases
S2851	Baricitinib	JAK
S2919	IOX2	HIF
S3147	Entacapone	others
S4246	Tranylcypromine (2-PCPA) HCl	MAO
S7062	EPZ5676	Methyltransferase
S7070	GSK J4 HCl	others
S7079	SGC 0946	Histone Methyltransferase
S7088	UNC1215	Epigenetic reader domain
S7110	(+)-JQ1	BET
S7113	Zebularine	DNA Methyltransferase
S7120	3-Deazaneplanocin A	Histone Methyltransferase
S7152	C646	Histone Acetyltransferase
S7189	I-BET-762	Epigenetic reader domain

Catalog Number	Product Name	Target
S7229	RGFP966	HDAC
S7231	*GSK2801*	Epigenetic reader domain
S7233	Bromosporine	Epigenetic reader domain
S7234	IOX1	Histone demethylases
S7237	OG-L002	Histone demethylases
S7256	SGC-CBP30	Epigenetic reader domain
S7265	MM-102	Histone Methyltransferase
S7276	SGI-1027	DNA Methyltransferase
S7281	*JIB-04*	Histone demethylases
S7292	RG2833 (RGFP109)	HDAC
S7294	*PFI-2*	Histone Methyltransferase
S7295	RVX-208	Epigenetic reader domain
S7304	CPI-203	Epigenetic reader domain
S7315	*PFI-3*	Epigenetic reader domain
S7324	TMP269	HDAC
S7353	EPZ004777	Histone Methyltransferase
S7360	OTX015	BET
S7373	UNC669	Epigenetic reader domain
S7438	ME0328	PARP
S7473	Nexturastat A	Nexturastat A
S7476	MG149	Histone Acetyltransferase
S7555	4SC-202	HDAC
S7570	UNC0379	Histone Methyltransferase
S7572	A-366	Histone Methyltransferase
S7574	GSK-LSD1 2HCl	Histone Demethylase
S7581	GSK J1	Histone Demethylase
S7582	Anacardic Acid	Histone Acetyltransferase
S7591	BRD4770	Histone Methyltransferase
S7610	UNC0631	Histone Methyltransferase
S7611	EI1	Histone Methyltransferase
S7616	CPI-169	Histone Methyltransferase
S7618	MI-2 (Menin-MLL Inhibitor)	Histone Methyltransferase
S7619	MI-3 (Menin-MLL Inhibitor)	Histone Methyltransferase
S7620	GSK1324726A (I-BET726)	Epigenetic reader domain
S7641	Remodelin	Histone Acetyltransferase
S7656	CPI-360	Histone Methyltransferase
S7680	SP2509	Histone Demethylase
S7681	OF-1	Epigenetic reader domain
S7748	EPZ015666	Histone Methyltransferase
S7795	ORY-1001 (RG-6016)	Histone Demethylase
S7796	GSK2879552 2HCl	Histone demethylase
S7804	*GSK503*	Histone Methyltransferase

Catalog Number	Product Name	Target
S7805	EPZ011989	Histone Methyltransferase
S7832	SGC707	Histone Methyltransferase
S7835	I-BRD9	Epigenetic reader domain
S8001	Rocilinostat (ACY-1215)	HDAC
S8006	BIX01294	Methyltransferase
S8049	Tubastatin A	HDAC
S8056	lomeguatrib	DNA Methyltransferase
S8111	GSK591	Histone Methyltransferase
S8112	MS023	Histone Methyltransferase
S8179	BI-7273	Epigenetic reader domain
S8180	PF-CBP1	Epigenetic reader domain
S8209	HLCL-61	Histone Methyltransferase
S8323	ITSA-1(ITSA1)	HDAC

Supplementary Methods

Cell culture. A549 and NCI-H1299 cells were maintained in DMEM, NCI-H446 cells were maintained in RPMI 1640 medium, and IMR-90 cells were maintained in MEM containing 0.1 mM NEAA, 1 mM sodium pyruvate, and 2 mM L-glutamate. BEAS-2B cells were maintained in BEBM with growth factors (Lonza, CC-4175). The DMEM, RPMI 1640, MEM and BEBM contained 10% FBS (HyClone), and 1% pen/strep (Invitrogen, Carlsbad, CA, USA). The cell lines were cultured at 37°C in an atmosphere of 5% CO_2.

Isolation, growth curves and immortalization of MEF cells. Mice were sacrificed at day 13.5 of pregnancy by cervical dislocation. The body was disinfected with 75% ethanol. Then, the uterine horns were dissected and washed in PBS, and each embryo was separated from its placenta and embryonic sac. The uterus was cut open along the uterine membrane for removal. The embryos were rinsed with PBS and digested in 0.25% trypsin containing 0.02% EDTA overnight at 4°C. Next, the cells were centrifuged at 1500 × *g* for 5 min and washed with DMEM three times. The MEF cells were maintained in DMEM containing 10% FBS, 1% pen/-strep, 0.1 mM NEAA, and 2 mM L-glutamate.

The $p53^{f/f}$, $p53^{f/f}$; *UBC*-Cre ($p53^{\Delta/\Delta}$), $Men1^{f/f}$, and $Men1^{f/f}$; *UBC*-Cre ($Men1^{\Delta/\Delta}$) MEF genotypes were identified by PCR. The MEF cells were induced with 1 μM TAM to generate primary $Men1^{f/f}$ and $Men1^{\Delta/\Delta}$ MEF cells, named passage 1 (P1) cells. For growth curves, primary $Men1^{f/f}$ or $Men1^{\Delta/\Delta}$ MEF cells at passage 2 (P2) (1.5×10^5) were seeded in a 6 cm dish and counted, and 1.5×10^5 cells were reseeded every 3 days. The growth curves show the cumulative numbers of cells. Representative examples of at least two independent experiments are shown in the growth curves. Isolated $p53^{f/f}$, $p53^{\Delta/\Delta}$, $Men1^{f/f}$ and $Men1^{\Delta/\Delta}$ MEF cells at P2 were seeded, infected twice with pGMLV-SV40T lentiviral particles, and subculture to established immortalized MEF cells. Subsequently, the cells were induced with 1 μM TAM to establish stable immortalized $p53^{f/f}$, $p53^{\Delta/\Delta}$, $Men1^{f/f}$ and $Men1^{\Delta/\Delta}$ MEF cell lines, which were used for subsequent experiments unless otherwise specified.

Targeted knockdown (KD) of genes with siRNA or shRNA. siRNA specifically

targeting *MEN1* was synthesized by Sigma, and the sequences are listed in table 2. IMR-90 cells were transfected with 50 nM siRNA using X-tremeGENE siRNA Transfection Reagent (Roche) according to the manufacturer's instructions. For stable A549 cell clones with *MEN1* or *p53* KD, retroviral packaging GP2-293 cells were transiently transfected with sh*MEN1* or sh*p53* retroviral plasmids using polyethyleneimine (PEI), and the culture supernatants containing retroviruses were collected 48 hours after transfection. A549 cells were infected with sh*MEN1* or sh*p53* retrovirus supernatants in the presence of 10 μg/ml polybrene and selected with 1 μg/ml puromycin for 2 weeks. Cells infected with retroviruses expressing sh*Luciferase* (sh*Luc*) plasmids were used as controls. To produce *MEN1*-overexpressing cell lines, A549, NCI-H446 and BEAS-2B cells were infected with retrovirus particles expressing pLNCX2-*MEN1* plasmids in the presence of 10 μg/ml polybrene and selected with 1 mg/ml G418 for 2 weeks. Cells infected with retroviruses expressing pLNCX2 plasmids were used as controls.

Preparation of histones. Cells (5×10^6) were harvested, and then 600 μl of buffer C (20 mM HEPES pH 7.9, 0.1% Triton X-100, 1.5 mM MgCl2, 1 mM PMSF, and 1 mM DTT) containing 1 μg/ml leupeptin, 1 μg/ml pepstatin and 1× phosphatase inhibitor was added. The cells were incubated on ice for 30 min. The lysates were then centrifuged at 2000 × *g* for 10 min. The pellets were washed once in buffer C and suspended in 600 μl of buffer D (10 mM HEPES pH 7.9, 1.5 mM MgCl2, 10 mM KCl, 1 mM DTT, and 1 mM PMSF) containing 1 μg/ml leupeptin, 1 μg/ml pepstatin, and phosphatase inhibitor. Sulfuric acid (2.0 N) was added to a final concentration of 0.4 N. The samples were incubated on ice for 45 min and centrifuged at 15000 × *g* for 10 min. A 1/5 volume of ice-cold 100% trichloroacetic acid (TCA) was added to the supernatant, and the mixtures was incubated for 45 min on ice. The precipitates were collected by centrifugation at 15000 × *g* for 10 min and washed sequentially with ice-cold 0.1% HCl and acetone. The pellets were air-dried and resuspended in RIPA buffer as the histone extract.

Ionizing radiation (IR) treatment. The mice harboring a TAM-inducible $Men1^{f/f}$; *UBC*-Cre *KO* allele were induced with 100 mg/kg TAM once a day for five days to generate *Men1 KO* ($Men1^{\Delta/\Delta}$) mouse models at 4 to 6 weeks of age. $Men1^{f/f}$ mice were considered WT mice. $Men1^{f/f}$ and $Men1^{\Delta/\Delta}$ mice were exposed to 3 Gy of IR via an

X-ray irradiator (RS2000, RAD SOURCE) at a dose rate of 2.03 Gy/min and sacrificed at 1 hours after IR. Lung tissues were fixed in 4% paraformaldehyde (PFA) for *IF assays*. For *in vitro* assays, cells were cultured in 10 cm dishes for 24 hours and exposed to the indicated doses of IR before being harvested at the indicated time points for follow-up experiments as described in each figure legend.

SA-β-gal staining. Senescent cells were detected with a SA-β-gal Staining Kit according to the manufacturer's protocol. Fifty fields were selected, and SA-β-gal-positive cells were counted. For animal samples, lung fragments were fixed in 4% PFA, dehydrated in 30% sucrose in PBS, and mounted in optimum cutting temperature (O.C.T.) compound (Tissue-Tek, #4583). Frozen sections of 5 μm thickness were cut, fixed in 1% PFA for 10 min at 4°C, washed in PBS and permeabilized with a solution of 2 mM MgCl2, 0.02% Nonidet P40 (NP-40) substitute, and 0.01% deoxycholic acid sodium salt in PBS. Incubation with SA-β-gal staining solution (included in the SA-β-gal Staining Kit) was performed at 37°C overnight. Sections were then counterstained with eosin according to standard protocols and viewed under a brightfield at 400× magnification.

Immunofluorescence (IF). Cells were seeded, fixed, permeabilized, blocked, and incubated with primary antibodies. Next, the cells were incubated with fluorophore-conjugated secondary antibodies and stained with 4', 6-diamidino-2-phenylindole (DAPI). For paraffin-embedded lung tissues, the sections were deparaffinized, rehydrated and thoroughly washed. After antigen retrieval, the sections were permeabilized with 0.3% Triton X-100 for 20 min, blocked with 5% nonimmune serum for 2 hours at room temperature and incubated with primary antibody as described above. Images were acquired with a confocal microscope (Olympus FV1000). Analysis of menin colocalization with γH2AX was performed with Olympus FV10-ASW software.

Measurement of endogenous reactive oxygen species (ROS). ROS production was performed using a cellular ROS detection assay kit, according to the manufacturer's specifications. Briefly, *Men1*$^{f/f}$ and *Men1*$^{\Delta/\Delta}$ MEF cells was irradiated with 3 Gy of IR and cells were then analyzed 6 hours post irradiation. Cells were counted and 1.0×10^{5} cells were incubated with 20 μM of the cell permeable fluorogenic

dye 2′,7′-dichlorofluorescin diacetate (DCFDA) for 30 min at 37°C in the dark, prior to flow cytometry analysis. The oxidative stress inducer, tertiary-butyl hydroperoxide (TBHP at 100 μM) was used as a positive control and it was added into cells for 4 hours before incubation with DCFDA, prior to analysis. Data were collected using Beckman Cytoflex S, and analyzed using Cytoflex S software.

Real-time quantitative PCR (RT-qPCR). RNA preparation was performed according to Trziol reagent, cDNA synthesis was carried out according to reverse transcription kit's instructions, and RT-qPCR was performed using an ABI PRISM 7300 detection system with the primers listed in table 2. RT-qPCR was repeated at least three times.

Immunohistochemistry (IHC). Tissues were fixed in 4% paraformaldehyde overnight and embedded in paraffin. 5 μm paraffin embedded sections were first deparaffinized in xylene. Gradient alcohol rehydration from 100% to 70%. Antigen retrieval was performed by boiling the slides in citrate buffer (10 mM, pH 6.0) in a water bath for 15 min in a pressure cooker, and allowed to cool to room temperature. Endogenous peroxidase was blocked by incubation with 0.3% hydrogen peroxide for 10 min. Slides were washed in PBS and blocked for 30 min with 5% bovine serum albumin (BSA). Slide were incubated overnight at 4°C with primary antibodies, followed by 1 hour with secondary antibody and Labelled Polymer-HRP at room temperature. Negative controls were treated identically, but without primary antibody. Subsequently, slides were stained with DAB Chromogen following manufacturer's instructions. Slides were counterstained with hematoxylin. After dehydration by alcohol, vitrification by xylene and neutral resin seals, slides were observed under microscope and photographed. For automatic quantification, digital images of stained tissues were captured with a Motic microscope using DigiLabII-Client software. Quantification of IHC staining was calculated by image pro plus software. The same index for the same batch of samples uses the unified quantification parameters. All of the indexes were measured by positive area, the parameter as (H: 0-30, S: 0-180, I: 0-180 or H: 0-20, S: 0-160, I: 0-160). For automatic quantification the area of positive cells per sample, we randomly analyzed three fields (400 times magnification, 800x600 pixel) per individual sample, then calculated the mean value for each sample. The mean value and stand error of mean were used to calculate for each group. Significance determined by Two-tailed

unpaired t tests. For panoramic scan of immunohistochemistry, the Motic microscope and Motic DSAssistant lite software were used.

Tumorigenesis assay *in vivo*. $5x10^6$ A549 sh*Luc* or A549 sh*MEN1* human lung adenocarcinoma cells mixed with matrigel (1:1) were injected subcutaneously into the flanks of 8-week male nude mice. Tumor size was measured every 3 to 5 days with a caliper, and the tumor volume was determined with the formula: Length x $Width^2$ x 0.5. 25 mg/kg SP2509 in 90% corn oil containing 10% DMSO was intraperitoneally injected twice a week for 3 weeks after the tumors have grown to 100-200 mm^3. The mice were euthanized after SP2509 treated for 3 weeks. The xenograft tumors were dissected and their weights were measured.

Supplementary Table 3. Key resources table

REAGENT or RESOURCE	SOURCE	IDENTIFIER
**Antibodies**		
**Antibodies/western blotting**		
β-actin	Cell Signaling	3700; RRID: AB_2242334
menin	Bethyl Laboratories	A300-105A; RRID: AB_2143306
p53	Cell Signaling	2524; RRID: AB_331743
p53	Santa Cruz	sc-126; RRID: AB_628082
Phosoho-p53 (Ser15)	Cell Signaling	9284; RRID: AB_331464
Phosoho-Rb (Ser780)	Cell Signaling	3590S; RRID: AB_2177182
MDM2	Abcam	ab 226939; RRID: AB_2754988
MDMX	Abcam	ab222905; RRID: AB_2754989
β-TrCP	Cell Signaling	4394S; RRID: AB_10545763
Skp1	Cell Signaling	12248; RRID: AB_2754993
APC2	Cell Signaling	12301; RRID: AB_2754994
Cullin1	Cell Signaling	4995; RRID: AB_2261133
γH2AX (Ser139)	Novus	NB100-78356; RRID: AB_1084673
RAD51	Novus	NB100-148; RRID: AB_10002131
53BP1	Novus	NB-100-305; RRID: AB_10001695
Ku70	Cell Signaling	4588S; RRID: AB_11179211
Ku80	Cell Signaling	2753S; RRID: AB_2257526
DNA-PKcs	Abcam	ab 32566; RRID: AB_731981
ATM	Abcam	ab78; RRID: AB_306089
Phosoho-ATM (Thr1981)	Abcam	ab81292; RRID: AB_1640207
ATR	Cell Signaling	2790; RRID: AB_2227860
Phosoho-ATR (Thr1989)	Cell Signaling	58014S; RRID: AB_2722679
CHK1	Cell Signaling	2360S; RRID: AB_563659
Phosoho-CHK1 (Ser345)	Cell Signaling	2348S; RRID: AB_331212
CHK2	Cell Signaling	2662S; RRID: AB_2080793
Phosoho-CHK2 (Thr68)	Abcam	Ab3501; RRID: AB_449196
Ubiquitin	Cell Signaling	3936; RRID: AB_331292
Histone 3	Abcam	ab1791; RRID: AB_302613
H3K4me1	Millipore	07-436; RRID: AB_310614
H3K4me2	Millipore	07-030; RRID: AB_10099880
H3K4me3	Millipore	17-614; RRID: AB_11212770
H3K9me3	Abcam	ab 8898; RRID: AB_306848
**Antibodies/IP**		
MDM2	Abcam	ab226939; RRID: AB_2754988
p53	Cell Signaling	2524; RRID: AB_331743
**Antibodies/IF**		
menin	Bethyl	A300-105A; RRID: AB_2143306
γH2AX (Ser139)	Millipore	05-636-I; RRID: AB_2755003

**Antibodies/ChIP**		
menin	Bethyl Laboratories	A300-105A; RRID: AB_2143306
H3K4me3	Millipore	17-614; RRID: AB_11212770
IgG	Millipore	2295402; RRID:
**IHC**		
Ki67	Abcam	ab66155; RRID: AB_1140752
NSE	Abcam	ab79757; RRID: AB_1603753
CgA	Abcam	ab15160; RRID: AB_301704
SPC	Millipore	ABC99; RRID: AB_2755001
menin	Bethyl	A300-106A; RRID: AB_162710
NCAM1	Proteintech	14255-1-AP; RRID: AB_2149421
ZEB1	Abcam	ab87280; RRID: AB_2040541
Syn	Abcam	ab14692; RRID: AB_301417
ASCL1	Abcam	ab38556; RRID: AB_722910
NEUROD1	Abcam	ab109224; RRID: AB_10861489
Phosoho-Rb (Ser780)	Abcam	ab32513; RRID: AB_777635
MYC	Abcam	ab39688; RRID: AB_731661
Vimentin	Abcam	ab45939; RRID: AB_2257290
Nestin	Abcam	ab27952; RRID: AB_776698
E-cadherin	Cell Signaling	3195S; RRID: AB_2291471
p53	Abcam	ab131442; RRID: AB_11155283
γH2AX	Cell Signaling	2577; RRID: AB_2118010
p16	Abcam	ab51243; RRID: AB_2059963
CGRP	Abcam	ab47027; RRID: AB_1141573
CD31	Abcam	ab28364; RRID: AB_726362
F4/80	Abcam	ab100790; RRID: AB_10675322
H3K9me3	Abcam	ab8898; RRID:AB_306848
MDM2	Abcam	ab226939; RRID: AB_2754988
**Antibodies/Biological Samples**		
NSCLC samples (n=157)	Tissue Bank, China-Japan Union Hospital of Jilin University	N/A
SCLC samples (n=34)	Tissue Bank, China-Japan Union Hospital of Jilin University	N/A
**Chemicals, Peptides, and Recombinant Proteins**		
Tamoxifen	Sigma	T5648-5G
Corn oil	Sigma	C8267
pGMLV-SV40T lentiviral	Genemeditech	GM-0220SV01
RIPA lysis buffer	Beyotime	P0013B
NP-40 lysis buffer	Beyotime	P0013F

**Chemicals, Peptides, and Recombinant Proteins**		
MG132	Selleck	S2619
Cycloheximide (CHX)	Sigma	C7698-1g
Polyethyleneimine (PEI)	Polyscences	23966-1g
Puromycin	Sigma	P9620
Polybrene	Sigma	H9268
G418	Amresco	Z859
Phosphatase inhibitor	Roche	04906837001
DAPI	Vector	H-1200
**Critical Commercial Assays**		
CCK8 kit	OBIO, China	OCPA(C)20132001
SA-β-gal Staining kit	Beyotime, China	C0602
ChIP kit	Millipore	17-295
RT-PCR kit	Takara	RR047A
IHC kit	Maxim, China	9370, 9708
DAB staining kit	Maxim, China	2031
DCFDA cellular ROS detection assay kit	Abcam	Ab113851
**Experimental Models: Cell Lines**		
Mouse: MEFs	This paper	N/A
Human: NSCLC cell line A549	ATCC	CCL-185
Human: NSCLC cell line H1299	ATCC	CRL-5803
Human: SCLC cell line NCI-H446	ATCC	HTB-171
Human: fetal lung fibroblasts cell line IMR-90	Stem Cell Bank, Chinese Academy of Sciences	SCSP-5013
Human: GP2-293	Takara	631530
Human: normal bronchus cell line BEAS-2B	BeNa Culture Collection	ATCC/CRL-9609
**Experimental Models: Organisms/Strains**		
Mouse: *Men1*$^{+/-}$: 129S6/SvEvTac	(Crabtree et al., 2001)[1]	N/A
Mouse: *Men1*$^{f/f}$:129S(FVB)-*Men1*$^{tm1.2Ctre}$/J	Jackson Laboratory	005109; RRID: IMSR_JAX:005109
Mouse: *MLL*$^{f/f}$: C57B6-SJL	(Craig et al., 2007)[2]	N/A
Mouse: *Kras*$^{LSL\text{-}G12D/+}$: B6.129S4-*Kras*^{tm4Tyj}/NJU	Model Animal Research Center of Nanjing University	N/A
Mouse: *UBC*-cre: B6;129S-Tg(*UBC*-cre/ERT2)1Ejb/J	Jackson Laboratory	007001; RRID: IMSR_JAX:007001
Mouse: *Sftpc*-cre: B6.129S-*Sftpc*$^{tm1(cre/ERT2)B1h}$/J	Jackson Laboratory	028054; RRID: IMSR_JAX:028054

**Plasmids and Virus Strains**		
pLNCX2-*MEN1*	Takara	631503
Retrovirus-expressing sh*MEN1*	Takara	631530
Retrovirus-expressing sh*p53*	This paper	N/A
Retrovirus-expressing *Kras*^{G12D}	This paper	N/A
Software and Algorithms		
Origin 8.0	OrigiLab	https://www.originlab.com/index.aspx?go=Company/NewsAndEvents/PressRoom&pid=1032

Supplementary Table 4. The primers or siRNA sequence used in the article

**RT-qPCR**		
H-*β-actin*	Forward	5'-TCAGAAGGATTCCTATGTGGGCGA-3'
	Reverse	5'-TTTCTCCATGTCGTCCCAGTTGGT-3'
H-*MEN1*	Forward	5'-ATCACAGGCACCAAATTGGACAGC-3'
	Reverse	5'-AACACTACCCAGGCATGATCCTCA-3'
H-*p53*	Forward	5'-GCCGTCCCAAGCAATGGATGATTT-3'
	Reverse	5'-TCTGGCATTCTGGGAGCTTCATCT-3'
H-*MDM2*	Forward	5'-TTCGTGAGAATTGGCTTCC-3'
	Reverse	5'-GGCAGGGCTTATTCCTTTTCT-3'
H-*βTrCP*	Forward	5'-CCTGGATGCCAAATCACTATG-3'
	Reverse	5'-TCCTCTTCGTTCTGCCAG-3'
H-*IL-6*	Forward	5'-AAATTCGGTACATCCTCGACGGCA-3'
	Reverse	5'-AGTGCCTCTTTGCTGCTTTCACAC-3'
H-*IL-1β*	Forward	5'-CATGGGATAACGAGGCTTATGT-3'
	Reverse	5'-CCCAAGGCCACAGGTATTT-3'
H-*p16*	Forward	5'-GGTCGGGTAGAGGAGGT-3'
	Reverse	5'-CCCATCATCATGACCTGGAT-3'
H-*Rb*	Forward	5'-ACTGAAAAGAGTGAAGGATATAGG-3'
	Reverse	5'-GGTAATACAAGCGAACTCCAAG-3'
M-*β-actin*	Forward	5'-CTAAGGCCAACCGTGAAAAG-3'
	Reverse	5'-ACCAGAGGCATACAGGGCA-3'
M-*p53*	Forward	5'-GGAACTCTCTTCCCTCACATTC-3'
	Reverse	5'-TCCTGCCTCAACTGTCTCTA-3'
M-*MDM2*	Forward	5'-GAAAAGCCTGAGGCTGGTAGAA-3'
	Reverse	5'-AACATAGGCAACCACCAGGAA-3'
M-*βTrCP*	Forward	5'-CAAAAGGATAGTGAGCGGAG-3'
	Reverse	5'-AAACTGGAGGCGGAAAAC-3'
M-*Skp1*	Forward	5'-TGATGTCACATGCAAGACTG-3'
	Reverse	5'-GCAGTTCACTTCTCTTCACAC-3'
M-*RBX1*	Forward	5'-GGATCTTTGTATCGAATGTCAGG-3'
	Reverse	5'-CGTTTTGAGCCATCGAGAG-3'
M-*APC2*	Forward	5'-GCTCTAAATGACCCCAAGATG-3'
	Reverse	5'-CGACCACAAGCCTTATCAAG-3'
M-*Cullin1*	Forward	5'-GCTCTAAATGACCCCAAGATG-3'
	Reverse	5'-CGACCACAAGCCTTATCAAG-3'
M-*Cullin3*	Forward	5'-GCAACATCTACAGGCAACG-3'
	Reverse	5'-ACTGAGTAGGCCAATATCCC-3'
M-*PCAF*	Forward	5'-AACCCTAACCCCTCTCCTAC-3'
	Reverse	5'-CTCTTCCTCTGACACATTCTCC-3'
M-*HAUSP7*	Forward	5'-GGGTTTCCACAAGATCAGATTC-3'
	Reverse	5'-GCCGTCAGCTTCATTATCTAAC-3'
M-*USP2a*	Forward	5'-CTGTATGCTGTGTCCAATC-3'
	Reverse	5'-GGAGTCATTGAAAGTGTGCC-3'

**RT-qPCR**		
M-*USP15*	Forward	5'-GGGCTGGAATAAACTTGTCAG-3'
	Reverse	5'-GTTCTCACAGAGCTTCAGTTC-3'
M-*p16*	Forward	5'-GCTGGGTGGTCTTTGTGTA-3'
	Reverse	5'-TTAGCTCTGCTCTTGGGATTG-3'
M-*IL-6*	Forward	5'-GGATGTCTGTAGCTCATTCTG-3'
	Reverse	5'-CAACTGGATGGAAGTCTCTTG-3'
M-*IL-8*	Forward	5'-GTCCTTAACCTAGGCATCTTCG-3'
	Reverse	5'-TCTGTTGCAGTAAATGGTCTCG-3'
M-*IL-1β*	Forward	5'-GGTGTGTGACGTTCCCATTA-3'
	Reverse	5'-ATTGAGGTGGAGAGCTTTCAG-3'
M-*TGF-β*	Forward	5'-ACCTGGGTTGGAAGTGGAT-3'
	Reverse	5'-GAAGCGCCCGGGTTGTGTTGGTT-3'
**ChIP**		
M-*βTrCP*-1	Forward	5'-GCTCTGTAGACAAGACTAGCC-3'
	Reverse	5'-GCTTTTAGTCCCAACACTTAGG-3'
M-*βTrCP*-2	Forward	5'-TGAGTTCTATCTGTCCCTGG-3'
	Reverse	5'-TGCAAGATTCCGATAACATCC-3'
M-*βTrCP*-3	Forward	5'-GCTTTGCCTTCTTAGAGACTG-3'
	Reverse	5'-GCCAAATGTAGAGCACTCG-3'
M-*βTrCP*-4	Forward	5'-CGACTCTACGCAGTGACC-3'
	Reverse	5'-ACTTGTAGTTCTGGAGGCTC-3'
M-*p16*-1	Forward	5'-CAGCTTCTAATCCCAGCAGTTA-3'
	Reverse	5'-GAGACAGACAGAGACAGAGAGA-3'
M-*p16*-2	Forward	5'-GGGAGACAGAGTTCTGAGTTTATG-3'
	Reverse	5'-TACTGGACAGAAGGGAGGATTTA-3'
**siRNA**		
MEN1	Forward	5'-GGAACCTGGCAGATCTAGA-3'
	Reverse	5'-TCTAGATCTGCCAGGTTCC-3'
βTrCP	Forward	5'-AAGUGGAAUUUGUGGAACAUC-3'
	Reverse	5-'UUCACCUUAAACACCUUGUAG-3'
NC	Forward	5'-UUCUCCGAACGUGUCACGUTT-3'
	Reverse	5'-ACGUGACACGUUCGGAGAATT-3'
**Genotype**		
Kras^{G12D}	Wild type	5'-GTCGACAAGCTCATGCGGG-3'
	Common	5'-CGCAGACTGTAGAGCAGCG-3'
	Mutant	5'-CCATGGCTTGAGTAAGTCTGC-3'
Men1 flox	Wild type	5'-TCCAGTCCCTCTTCAGCTTC-3'
	Mutant	5'-GCCATTTCATTACCTCTTTCTCCG-3'
	Common	5'-TACCACTGCAAAGGCCACGC-3'
Men1 deletion	Common	5'-CCCACATCCAGTCCCTCTTCAGCT-3'
	Floxed	5'-AAGGTACAGCAGAGGTCACAGAG-3'
	Deletion	5'-GACAGGATTGGGAATTCTCTTTT-3'

Genotype		
Sftpc-Cre	Mutant	5’-ACACCGGCCTTATTCCAAG-3’
	Common	5’-TGCTTCACAGGGTCGGTAG-3’
	Wild type	5’-CATTACCTGGGGTAGGACCA-3’
Men1$^{+/-}$	Common	5’-CCCACATCCAGTCCCTCTTCAGCT-3’
	Wild type	5’-CCCTCTGGCTATTCAATGGCAGGG-3’
	Mutant	5’-CATAAAATCGCAGCAGGTGGGCAA-3’
MLL flox	Forward	5’-TCTCTGAAGTAAGCCTTTCTTAG-3’
	Reverse	5’-CAGTGGACATTCCAACTCTTCAA-3’
MLL	Deletion	5’-CACCCAGCATTGCAGAGTCAG-3’
	Transgene	5’-GCGGTCTGGCAGTAA AAACTATC-3’
	Transgene	5’-GTGAAACAGCATTGCTGTCACTT-3’
UBC Cre	Forward	5’-CTAGGCCACAGAATTGAAAGATCT-3’
	Reverse	5’-GTAGGTGGA AATTCTAGCATCATC C-3’

Supplementary References

1. Crabtree, J.S. *et al.* A mouse model of multiple endocrine neoplasia, type 1, develops multiple endocrine tumors. *Proc Natl Acad Sci U S A* **98**, 1118-1123 (2001).
2. Jude, C.D. *et al.* Unique and independent roles for MLL in adult hematopoietic stem cells and progenitors. *Cell stem cell* **1**, 324-337 (2007).
